# Synaptic plasticity in a recurrent neural network for versatile and adaptive behaviors of a walking robot

**DOI:** 10.3389/fnbot.2015.00011

**Published:** 2015-10-13

**Authors:** Eduard Grinke, Christian Tetzlaff, Florentin Wörgötter, Poramate Manoonpong

**Affiliations:** ^1^Bernstein Center for Computational Neuroscience, Third Institute of Physics, Georg-August-Universität GöttingenGöttingen, Germany; ^2^Department of Neurobiology, Weizmann Institute of ScienceRehovot, Israel; ^3^Embodied AI and Neurorobotics Lab, Center for BioRobotics, The Mærsk Mc-Kinney Møller Institute, University of Southern DenmarkOdense M, Denmark

**Keywords:** neural dynamics, hysteresis, correlation-based learning, navigation, walking robots, autonomous robots

## Abstract

Walking animals, like insects, with little neural computing can effectively perform complex behaviors. For example, they can walk around their environment, escape from corners/deadlocks, and avoid or climb over obstacles. While performing all these behaviors, they can also adapt their movements to deal with an unknown situation. As a consequence, they successfully navigate through their complex environment. The versatile and adaptive abilities are the result of an integration of several ingredients embedded in their sensorimotor loop. Biological studies reveal that the ingredients include neural dynamics, plasticity, sensory feedback, and biomechanics. Generating such versatile and adaptive behaviors for a many degrees-of-freedom (DOFs) walking robot is a challenging task. Thus, in this study, we present a bio-inspired approach to solve this task. Specifically, the approach combines neural mechanisms with plasticity, exteroceptive sensory feedback, and biomechanics. The neural mechanisms consist of adaptive neural sensory processing and modular neural locomotion control. The sensory processing is based on a small recurrent neural network consisting of two fully connected neurons. Online correlation-based learning with synaptic scaling is applied to adequately change the connections of the network. By doing so, we can effectively exploit neural dynamics (i.e., hysteresis effects and single attractors) in the network to generate different turning angles with short-term memory for a walking robot. The turning information is transmitted as descending steering signals to the neural locomotion control which translates the signals into motor actions. As a result, the robot can walk around and adapt its turning angle for avoiding obstacles in different situations. The adaptation also enables the robot to effectively escape from sharp corners or deadlocks. Using backbone joint control embedded in the the locomotion control allows the robot to climb over small obstacles. Consequently, it can successfully explore and navigate in complex environments. We firstly tested our approach on a physical simulation environment and then applied it to our real biomechanical walking robot AMOSII with 19 DOFs to adaptively avoid obstacles and navigate in the real world.

## 1. Introduction

Living creatures, like insects, with their limited neural computing show impressive versatile and adaptive behaviors (Ritzmann and Büschges, 2007; Gruhn et al., 2011; Schütz and Dürr, 2011). For example, they can walk around their complex cluttered environment (Ritzmann et al., 2004) and, at the same time, avoid, or climb over obstacles as well as escape from corners or deadlocks (Watson et al., 2002; Awe, 2008; Baba et al., 2010). By doing so, they can effectively explore the environment and successfully navigate through it. They solve the tasks by interacting with the environment and using their adaptive neural circuits (Heisenberg, 1998; Wessnitzer and Webb, 2006; Fuchs et al., 2010) in their sensorimotor loop to process sensory information and generate adequate motor commands (Strausfeld, 1999). Specifically, they use their exteroceptive sensors (e.g., antennae Burdohan and Comer, 1990; Okada and Toh, 2000; Comer et al., 2003) to detect environmental changes or obstacles and process this sensory information in higher brain areas by using the full capacity of their neural dynamics adapted by synaptic plasticity (Strausfeld, 1999; Wessnitzer and Webb, 2006; Tomchik and Davis, 2009; Guo et al., 2012; Frank et al., 2013). The processed information is then transmitted as descending signals to move their biomechanical legs through their neural locomotion control (Pearson et al., 1973) in the thoracic ganglia (Schaefer and Ritzmann, 2001; Ridgel et al., 2007; Bender et al., 2010). This shows that the ability to perform versatile and adaptive behaviors requires a combination of several components as neural dynamics, synaptic plasticity, sensory feedback, and biomechanics.

Generating such complex autonomous behaviors (walking, avoiding obstacles, escaping from corners/deadlocks, as well as exploring and navigating in an unknown cluttered environment) as insects, for artificial many degrees-of-freedom (DOFs) systems (like, legged robots) is still a challenging task. Attempts try to find solutions to the problem by using different approaches. Many of them use the conventional behavior-based robotics method, also known as subsumption architecture (Brooks, 1989; Wettergreen et al., 1995; Luk et al., 1996; Celaya and Porta, 1998; Celaya and Albarral, 2003), where a complex behavior is generated by combining several simple reactive behaviors. Each behavior is typically controlled by one reactive control unit and all units are run in parallel. For example, using this method, the physical six-legged walking machine Genghis (Brooks, 1989) can walk over rough terrain, avoid obstacles, and follow a person. While this behavior-based robotics method can achieve versatile behaviors, it still lacks learning ability to be adaptive and efficient in an unstructured complex environment. When facing corners or an insurmountable obstacle (like, wall), the behavior-based control method can drive a correct behavior only after the corner or obstacle is detected. If additional sensing and online learning (learning during behaving) are applied (Togelius, 2004), a robot can learn and adapt its movements properly (e.g., react earlier); thereby leading to efficient maneuvers. Another shortcoming of the behavior-based robotics method is that it is difficult to predict the overall behavior of the system as the number of behavior and the interaction between them increase.

Thus, machine learning, like reinforcement learning (RL) (Barfoot et al., 2006; Erden and Leblebicioğlu, 2008) and evolutionary computing (EC) (Parker, 2005; Seljanko, 2011; Risi and Stanley, 2012), has become an attractive tool for including adaptivity into artificial systems. For example, Erden and Leblebicioğlu (2008) used RL to generate free gaits and also let a six-legged walking machine learn to achieve a continuous and stable walking pattern with five legs in an abnormal case. Bongard et al. (2006) used EC as an adaptive process to generate successful motor patterns for locomotion, before and after damage of a starfish-like walking machine. With this technique, if the machine has been damaged, it could sense the problem and adapt its locomotion to compensate for this. While such machine learning approaches allow for leaning and adaptation, they usually require a long learning process for (complex) behavior generation. Furthermore, an objective function (i.e., reward for RL or fitness function for EC) needs to be properly designed to archive a learning goal. Besides these approaches, artificial neural networks (ANNs Beer et al., 1997; Lewinger and Quinn, 2011; Von Twickel et al., 2012) appear more appropriate due to their intrinsically distributed architecture (Schilling et al., 2013) and their capability to integrate different learning mechanisms for different timescales of adaptivity (Filliat et al., 1999; Fischer et al., 2004; Valsalam and Miikkulainen, 2008; Steingrube et al., 2010). They also allow to develop a controller as a composition of different neural modules (Valsalam and Miikkulainen, 2008). Furthermore, ANNs with recurrent connections show a wide variety of different neural dynamics (oscillations, hystereses, chaotic patterns, fixed points, etc.) which can be exploited for signal processing and locomotion generation (Steingrube et al., 2010; von Twickel et al., 2011; Toutounji and Pasemann, 2014). According to this, many studies mainly employ ANNs for the purpose of locomotion (Beer et al., 1997; Valsalam and Miikkulainen, 2008; Lewinger and Quinn, 2011; von Twickel et al., 2011; Von Twickel et al., 2012; Schilling et al., 2013; Toutounji and Pasemann, 2014). For example, Beer et al. (1997) developed a distributed neural network controller of a six-legged walking machine for generating locomotion with reflex actions to deal with irregular, slatted, and compliant terrains. Lewinger et al. (Lewinger and Quinn, 2011) developed neurobiologically-based control for an adaptively walking hexapod robot. The control allows the robot to walk, cross a small gap, step over an small bump, and seek a goal. Von Twickel et al. (2012) developed neurocybernetic control for generating locomotion of four-, six-, and eight-legged modular robots. While all these neural control mechanisms can generate different locomotion modes, they do not have complete mechanisms for generating complex autonomous behaviors (i.e., walking, climbing, avoiding obstacles, escaping from corners/deadlocks, as well as exploring and navigating in an unknown complex cluttered environment). Only a few works used ANNs for walking and navigating in simple cluttered environments with a few obstacles (Filliat et al., 1999; Fischer et al., 2004). Usually, most developed neural mechanisms (Filliat et al., 1999; Fischer et al., 2004; Von Twickel et al., 2012; Toutounji and Pasemann, 2014) use evolutionary techniques to optimize neural parameters (i.e., synaptic weights and structures). Thus, they typically end up with complex massive recurrent connectivity structures which is difficult to understand and be mathematically analyzed. Furthermore, they require a very long learning process which is not practical for real robot implementation.

From this point of view, we present here modular neural mechanisms with synaptic plasticity where their functions can be analyzed and understood. The complete mechanisms with online adaptation are implemented on a real biomechanical walking robot with 19 DOFs. They can generate complex autonomous behaviors of the robot in complex environments with many obstacles, different sharp corners, and narrow passages. During exploring the environment, the robot has to learn to avoid different object arrangements by turning away in an appropriate manner. The neural mechanisms consist of two main components: (i) neural sensory processing with online learning and (ii) modular neural locomotion control. We use two ultrasonic sensors to detect the obstacles (exteroceptive feedback) and use the neural dynamics adapted by plasticity in the sensory processing network to process sensory signals and generate different behaviors (e.g., turnings, walking speeds, climbing) with short-term memory for the robot. The turning information is transmitted as descending steering commands to the neural locomotion control network previously developed by us Manoonpong et al. (2013). Finally, the outputs of the locomotion control network drive the biomechanical legs of the robot. This will result in versatile and adaptive abilities of the robot which include walking, avoiding or climbing over obstacles, escaping from corners/deadlocks and narrow passages, as well as exploring and navigating in complex unknown environments. Taking all these behaviors into account, this approach, following insects' strategy, basically combines neural dynamics, synaptic plasticity, sensory feedback, and biomechanics to achieve such complex autonomous behaviors.

The main contribution here is not only to demonstrate complex behaviors in complex environments but also to show an option of integrating synaptic plasticity in a small recurrent neural network to effectively exploit the rich neural dynamics (i.e., hysteresis effects and attractor dynamics) for generating complex behaviors in a sensorimotor loop of a behaving many DOFs system. The following section describes the adaptive neural sensory processing network developed in this study and is followed by a short descriptions of the neural locomotion control network and the biomechanical walking robot AMOSII (for further details please see Manoonpong et al., 2013). Note, for a better understanding several results are already provided in this section. The main experimental results are then presented in Section 3 and discussed in Section 4.

## 2. Materials and methods

**Figure 1:**
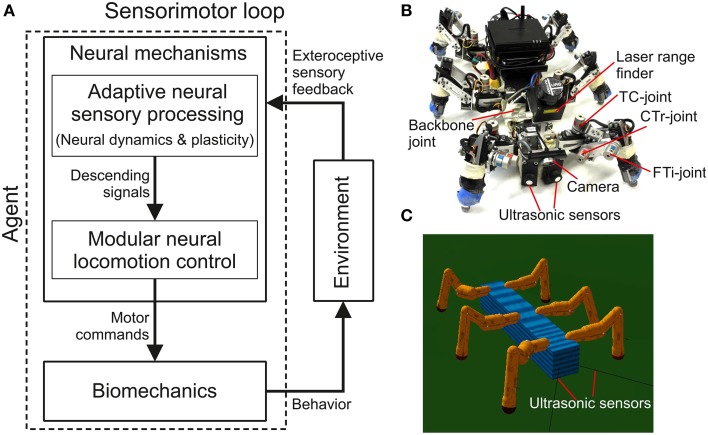
**Adaptive embodied neural closed-loop control setup and the real and simulated biomechanical walking robots AMOSII. (A)** The setup of adaptive embodied neural closed-loop control for complex autonomous behaviors with versatility and adaptivity. It consists of the adaptive neural sensory processing network with synaptic plasticity, the modular neural locomotion control network, the biomechanical walking robot AMOSII, and the environment. The sensory processing network is in the focus of this study while the locomotion control network and the robot have been developed earlier (Manoonpong et al., 2013). **(B)** The real biomechanical walking robot AMOSII with 19 DOFs and its sensors. It has one backbone joint for climbing and 3-jointed legs for walking on different terrains. Its two ultrasonic sensors installed at the front are used to provide exteroceptive sensory feedback to its adaptive neural sensory processing network. Additionally, a camera and a laser range finder are used for terrain recognition and obstacle height detection. **(C)** The simulated AMOSII using the LPZRobots simulation environment (Martius et al., 2010).

In this study complex autonomous behaviors with versatility and adaptivity of a walking robot are generated through a sensorimotor loop which involves neural dynamics, synaptic plasticity, sensory feedback, and biomechanics (Figure 1). Neural dynamics and plasticity are embedded in an adaptive neural sensory processing network (Figure 1A). The network with its online synaptic plasticity mechanism, which is the main contribution of this work, will be described in the first section and followed by a short description of the modular neural locomotion control network and the used biomechanical walking robot AMOSII (Figures 1B,C). Some results are described alongside the introduced components to provide a better understanding of their functionalities.

### 2.1. Adaptive neural sensory processing network

**Figure 2:**
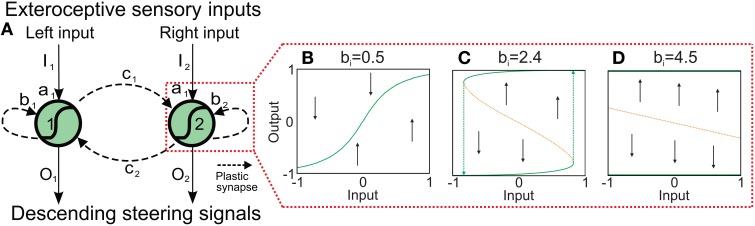
**Adaptive neural sensory processing network. (A)** The network is a two-neuron recurrent network based on the MRC network. Here, the synaptic weights of the network are adapted by an online correlation-based learning rule with synaptic scaling. Changing the connections also changes the neural dynamics of the network. Already for the single neuron with a recurrent connection *b* the possible neural dynamics are **(B)** an approximately linear *I*−*O*-relation, **(C)** a hysteresis, and **(D)** a two state (active, non-active) system. Obviously, the two-neuron system exhibits more complex dynamics. Green solid line: stable attractor; orange doted line; unstable attractor (for the influence of the inhibitory connections, see Supplementary Material).

Although a two-neuron recurrent network has a limited complexity it already shows a wide variety of interesting dynamical properties (Figure 2 and Supplementary Material) which can be exploited for sensory processing, state memorization, and behavior control (Hülse and Pasemann, 2002; Hülse et al., 2007; Manoonpong et al., 2010). Based on this network, Hülse et al. (Hülse and Pasemann, 2002) employed an evolutionary algorithm (Hülse et al., 2004) to evolve the optimal parameters of the system. The resulting network, named minimal-recurrent controller (MRC), consists of mutual inhibitory synapses between the neurons and a self-excitatory synapse at each neuron. This system exhibits a hysteresis in its neuronal activations (Figure 2C) which can be used to process sensory signals and generate obstacle avoidance behavior (tested on a simple two wheeled Khepera robot with two DOFs Hülse et al., 2004). However, using the evolved parameters of the synaptic weights or other fixed values, the robot can get stuck in complex environments composed of many obstacles, sharp corners, and narrow passages. Thus, we use the two-neuron network as our basic network structure (Figure 2) and adapt the synaptic weights by an unsupervised correlation-based learning rule with synaptic scaling (Tetzlaff et al., 2011) while interacting with the environment. This allows the system to use *all* possible neural dynamics (i.e., hysteresis, single fixed points, etc.) and, thereby, to adaptively avoid obstacles in a complex environment.

All neurons of the network are modeled as discrete-time non-spiking neurons. The neural outputs (*O*_1, 2_) are governed by Equations (1, 3), respectively:

(1) $$O_{1}(t+1)=\tanh\left[b_{1}O_{1}(t)+c_{2}O_{2}(t)+a_{1}I_{1}(t)\right],$$

(2) $$O_{2}(t+1)=\tanh\left[b_{2}O_{2}(t)+c_{1}O_{1}(t)+a_{1}I_{2}(t)\right],$$

where *b*_1, 2_ are the weights of the self-excitatory synapses, *c*_1, 2_ are the weights of inhibitory synapses between the neurons, and *I*_1, 2_ are the sensory input signals. In this study, two ultrasonic sensors, mounted on the forehead of the walking robot, provide the sensory input signals to the network. The sensors, acting as an insect' antennae, are for obstacle detection. The range of each sensor is adjusted to 40 cm. Before feeding the raw sensory signals to the network, we map them to the range of [−1, 1] where −1 means no obstacle in the range and 1 means that an obstacle is near (about 5 cm distance). The output signals (*O*_1, 2_ ∈ [−1, 1]) of the two-neuron network are transmitted to the neural locomotion control network triggering behavior performed by the biomechanics (Figure 1A). Thus, the robot can autonomously perform obstacle avoidance behavior through a sensorimotor loop with respect to environmental stimuli. In other words, it will walk around and also avoid obstacles if they are detected. Note that if the outputs *O*_1, 2_ are zero, the robot has actually to stop moving. However, this situation is very unlikely as the robot is, without external trigger, forced to move forward (*O*_1, 2_ = −1), otherwise the robot would stand still and do nothing. In Table 1 different walking directions with respect to the sign of the output signals of the sensory processing network are summarized.

**Table 1:** **Different walking directions with respect to the sensory processing outputs**.

**Output 1 (left, *O*_1_)**	**Output 2 (right, *O*_2_)**	**Walking direction**
Negative	Negative	Forward
Positive	Positive	Backward
Negative	Positive	Turn left
Positive	Negative	Turn right
Zero	Zero	Stop

*The amplitude determines the walking speed*.

#### 2.1.1. Non-plastic synapses

**Figure 3:**
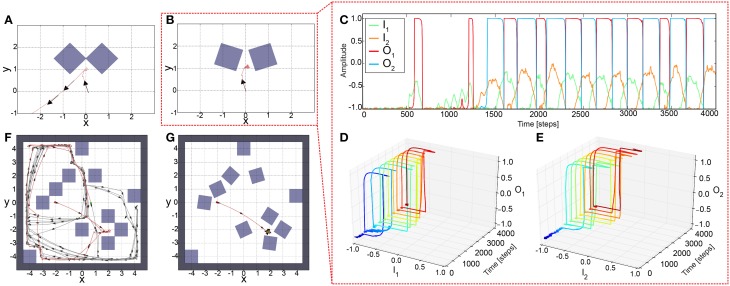
**Simulation results of AMOSII using non-plastic synapses in different deadlock situations. (A)** The robot successfully escaped from a simple deadlock situation. **(B)** If the situation becomes more complicated (only by inducing a small gap between obstacles) the robot is unsuccessful and trapped. However, the neuronal activations **(C)** during this unsuccessful escaping behavior follow the desired hysteresis between input and output (**D,E**; color code illustrates time from dark blue to dark red). Both tests were run for 5000 time steps. Similar effects arise in more complex environments. **(F)** In an environment with obstacles but no sharp corners and narrow passages the agent was able to explore the area without getting stuck (red line: initial 70, 000 time steps; gray line: further 330, 000 time steps). **(G)** In a more difficult environment with obstacles, sharp corners, and narrow passages the same network setup leads to an oscillating behavior as in **(B)** and the agent is trapped. Red dot (−2, 0) defines the starting position and green dot shows the position after 70, 000 time steps. Arrows indicate walking direction.

To understand neural dynamics and investigate the performance of the network with static, non-plastic synapses, we manually adjusted the synaptic weights (*b*_1, 2_ and *c*_1, 2_) for AMOSII on the basis of their well understood functionalities (Hülse and Pasemann, 2002; Manoonpong et al., 2008). The weights from the input to output units were set to *a*_1_ = 4.7. The self-connections (*b*_1, 2_) were tuned to derive a reasonable hysteresis interval on the input space (Figure 2C). The hysteresis assures that the system still turns although the sensors do not detect an obstacle anymore. Note, the width of the hysteresis is proportional to the strength of the self-connections. Thus, the hysteresis width determines the turning angle in front of the obstacles for avoiding them, i.e., the larger the hysteresis interval (distance between green arrows in Figure 2C), the larger the turning angle. Thus, the self-connections are set to 2.4 to obtain a suitable turning angle of AMOSII. A too small self-connection will not induce a hysteresis and, therefore, a long enough turning (Figure 2B) while a too strong connection will induce either no turning (non-active state) or permanent turning (active state; Figure 2D). Finally, the mutual connections (*c*_1, 2_) between the neurons are symmetrized and manually adjusted to −3.5. Such inhibitory recurrent connections form a so-called even loop (Wessnitzer and Webb, 2006). In an even loop, in general, only one neuron at a time is able to produce a positive output, while the other one has a negative output, and vice versa. This guarantees the optimal functionality for avoiding obstacles and escaping from corners. The hysteresis induced by the self-connection enables a strong activation even after turning away from one obstacle and, by inhibiting the other neuron, continues turning. Stucked in a trap, by this method the agent or robot can turn until it gets out. In other words, in a trap (e.g., Figure 3A) the system remembers for a certain duration the first object and executes the related behavior (turn) and, in addition, it does not trigger the behavior induced by the second object which would be a turning in the opposite direction. This network, with the best working configuration, enables AMOSII to avoid obstacles and escape from corners and deadlocks (Figures 3A,F). However, AMOSII got stuck when sharp corners or narrow passages are present (Figures 3B,G). The neural dynamics for a narrow passage (Figure 3B) are shown in (Figure 3C). Although the hysteresis effect is triggered (Figures 3D,E), the turning angle is in such a case not large enough and the behavior induced by the second object is also triggered (turn in the opposite direction). Now, again a hysteresis starts which is not long enough, too. Thus, the robot starts to oscillate between the objects and is trapped. To avoid this undesirable behavior the memory of the first obstacle has to longer dominate the dynamics of the system and, thereby, to increase the turning angle. Thus, the system needs a mechanism to modify the synaptic weights of the network during behavior, thereby, for instance, changing the neural dynamics of the network from an hysteresis effect (Figure 2C) to a single fixed point attractor having a prolonged memory (Figure 2D) and vice versa. Therefore, we introduce a biological reasonable plasticity rule based on the interaction between correlation-based learning and synaptic scaling (Tetzlaff et al., 2011) to adapt the synaptic weights in an online (while behaving) manner described in the following.

#### 2.1.2. Plastic synapses using correlation-based learning and synaptic scaling

To modify all synaptic weights (*b*_1, 2_ and *c*_1, 2_) while the agent interacts with the environment, we use here correlation-based learning (Kolodziejski et al., 2008) based on three factors: the output activity *O*_*i*_(*t*) of the network at the time step *t*, the output activity *O*_*i*_(*t*−1) of the network at the previous time step *t*−1, and a reflex signal *T*_*i*_(*t*) (*i* ∈ {1, 2}). The reflex signal is used to control the learning process which will start as soon as the robot detects an obstacle at close range (about 30 cm). Additionally, we also employ synaptic scaling (Tetzlaff et al., 2011, 2012) to stabilize the synaptic weights. To assure that the synaptic weights do not change their sign, for the learning rule, we map the outputs *O*_*i*_ ∈ [−1, 1] to the positive interval *v*_*i*_ ∈ [0, 1]. Thus, synaptic weights are updated as follows:

(3) $$b_{i}(t+1)\;=\mu_{b}\cdot v_{i}(t-1)\cdot v_{i}(t)\cdot T_{i}(t)+\gamma_{b}(k-v_{i}(t))\cdot b_{i}{\left(t\right)}^{2},$$

(4) $$q_{i}(t+1)\;=\mu_{q}\cdot v_{i}(t-1)\cdot v_{i}(t)\cdot T_{i}(t)+\gamma_{q}(k-v_{i}(t))\cdot q_{i}{\left(t\right)}^{2},$$

(5) $$c_{i}(t+1)=\frac{1}{2}\cdot \left(q_{1}(t+1)+q_{2}(t+1)\right).$$

μ_*b, q*_ are the learning rates or time scales of correlation-based learning which are set to μ_*b*_ = 0.0065, μ_*q*_ = 0.015, and γ_*q, b*_ are the forgetting rates or time scales of synaptic scaling which are set to γ_*q, b*_ = 0.0003. The learning and forgetting rates are empirically selected. Note, we introduce the auxiliary variables *q*_*i*_ for the inhibitory weights and calculate from them the average inhibitory weight *c* = *c*_1_ = *c*_2_ in order to maintain symmetric inhibition and, thereby, the even loop (Wessnitzer and Webb, 2006). The parameter *k* is an offset and set to *k* = −0.01. The reflex signal *T*_*i*_ is computed from the sensory input *I*_*i*_:

(6) $$T_{i}(t)\left(I_{i}(t)\right)=\{\begin{array}{cc} 1 & \text{if}I_{i}(t)>-0.5,\\ 0 & \text{if}I_{i}(t)\leq -0.5. \end{array}$$

In principle, this learning mechanism adapts the synaptic weights in a way that the synaptic weights and therefore the neural output will reach (via the hysteresis; Figure 2C) and stay (Figure 2D) at the upper fixed point while the robot is trying to escape from a narrow passage or deadlock. Thereby, the robot will escape from the situation by performing a very large turning angle. Once the robot has escaped or does not detect an obstacle any more, the second part of the mechanism (synaptic scaling) will decrease the synaptic weights such that the neural output returns to the lower fixed point (Figures 2B,C); thereby the robot will stop turning and continue to walk forward. In other words, the interaction of correlation learning and scaling moves the neural system between a two fixed point state (i.e., hysteresis effects) and single fixed point states and vice versa. Note that the used learning mechanism is independent of the used initial weight values (Tetzlaff et al., 2011, 2012).

**Figure 4:**
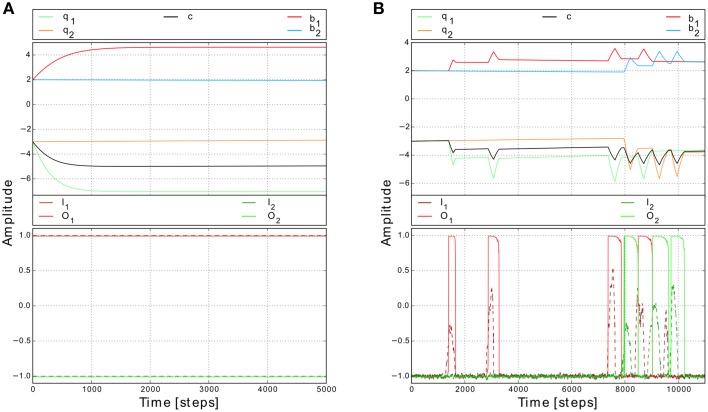
**Synaptic weight changes for different input signals. (A)** Weight changes for constant inputs (*I*_1_ = 1 and *I*_2_ = −1). While the input is active (i.e., here left input), the corresponding synaptic weights (i.e., here *b*_1_ and *c*_1_) increase and start to converge to certain values. Note, this value does not depend on the initial conditions (Tetzlaff et al., 2011). **(B)** Weight changes for changing inputs (*I*_1, 2_) from a simulated environment. Synaptic weights increase quite fast when the input is strong enough to trigger the reflex signal. Afterwards, if no input is present, the synaptic weights start to decay slowly.

To show the basic dynamics of this adaptive system we, first, initialized the synaptic weights with the values similar to the ones used for non-plastic synapses (i.e., *b*_1, 2_ = 2.4, *c*_1, 2_ = −3.5) and then provided constant inputs to the network (*I*_1_ = 1, *I*_2_ = −1). According to the input values, *T*_1_ is one and *T*_2_ is zero. The synaptic weights *b*_1_ and *c*_1_ increase and asymptotically converge while *b*_2_ and *c*_2_ remain unchanged (Figure 4A). Interestingly, if the system receives changing inputs, the weight changes are faster and “peak-like” (Figure 4B). Afterwards, if the input becomes inactive, the corresponding synaptic weights starts slowly to decay. Thus, this plasticity mechanism enables the system to reach the whole range of synaptic weight values.

**Figure 5:**
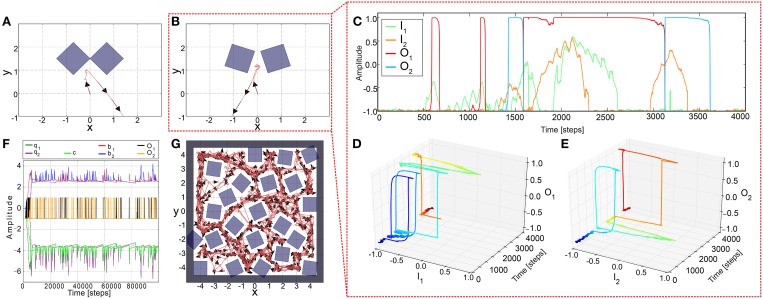
**Simulation results of AMOSII using plastic synapses in deadlock situations. (A)** The robot successfully escaped from a simple deadlock situation. **(B)** If the situation becomes more complicated (small gap between obstacles) the adaptive robot is successful and not trapped. Neuronal activations **(C)** are much more complicated due to the switch of the system between hysteresis and single attractor states (**D,E**; color code illustrates time from dark blue to dark red). Both tests were run for 5000 time steps. **(F,G)** In a more difficult environment with obstacles, sharp corners, and narrow passages, the adaptive robot is able to escape from all deadlocks, corners, etc. and explores the whole environment by permanently adapting the synaptic weights **(F)**. The experiment was run for 1,000,000 time steps.

To test the performance of the adaptive sensory processing network, we tested it, similar to the constant system, in two different environments (Figure 5). Remarkably, plasticity enables the robot to successfully escape from a simple deadlock (Figure 5A) and complex one (Figure 5B). For the complex case, after a few turns to and from, the robot was able to escape from it and was not trapped. The neural dynamics of the narrow passage experiment (Figure 5B) is depicted in (Figures 5C–E). This show that the learning mechanism is able to change the neural dynamics in a way that the neural output can stay at an upper fixed point for a long duration, thereby, implying a very large turning angle resulting to a successful escape from the difficult deadlock situation.

**Figure 6:**
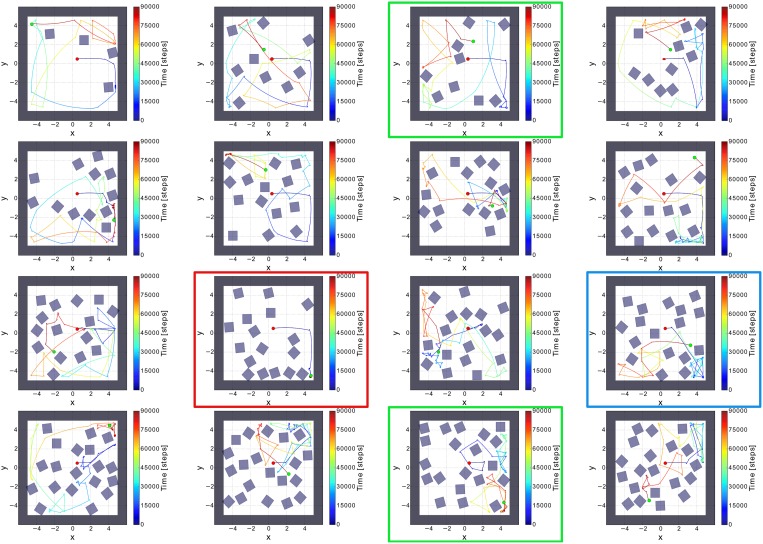
**Sixteen random environments with different numbers and orientations of obstacles**. The red dot is the starting point of the robot and the green dot indicates the end point of the robot. The environments were randomly filled with obstacles from the amount of 4–20 boxes. Red square shows the fail case. Green and blue squares show the success cases where the agent was facing to a confined space and a trap, respectively.

To evaluate the comprehensive performance of the network, as the parameters of the environments are huge, we tested 50 random environments consisting of different numbers and orientations of obstacles (i.e., non-climbable obstacles) and surrounding walls (see Figure 6). We varied the number of the obstacles from 2 to 20. According to this, the agent was facing different (random) situations like obstacles, sharp corners, deadlocks, and narrow passages. Remarkably, the agent failed only one to explore the whole environment (see Table 2). Very small confined spaces can pose a problem for the agent to escape because it is barely/not able to turn; thereby getting stuck (see red square in Figure 6). However, the agent can successfully escape if the confined space is a bit bigger than in the fail case (see green square in Figure 6) or even there is a trap (see blue square in Figure 6).

**Table 2:** **Success and failure rate of different tested situations**.

**Environments**	**Success**	**Failure**
50	49	1

### 2.2. Modular neural locomotion control network

The modular neural locomotion control network for locomotion generation of the biomechanical walking machine AMOSII consists of three main neural components (modules): central pattern generator (CPG)-based control module (Manoonpong et al., 2013), local leg control module (Manoonpong et al., 2013), and backbone joint control module (Goldschmidt et al., 2014). The CPG-based control basically coordinates all leg joints and generate multiple insect-like gaits and different walking directions (forward/backward walking, turning left, and right). The local leg control, relying on proprioceptive sensory feedback (like foot contact sensors) and internal forward models, adapt the movement of an individual leg of AMOSII for foothold searching and elevation, thereby, enabling rough terrain locomotion and supporting the body of AMOSII during climbing. The backbone joint control, using exteroceptive and proprioceptive feedback, generates the leaning movements of the backbone joint for climbing over obstacles. Here we will describe all these components in brief since they are not the main contribution here but they are required to support the adaptive neural sensory processing network (described above) for generating the versatile and adaptive behaviors of AMOSII.

The CPG-based control has four components: (1) a two-neuron CPG system with neuromodulation for generating different periodic patterns, (2) neural CPG postprocessing for shaping the CPG patterns to obtain smooth leg movements, (3) neural motor control consisting of two different feedforward neural networks [phase switching network (PSN) and velocity regulating networks (VRNs)] for controlling walking direction (forward/backward and turning), and (4) motor neurons with delay lines for sending motor commands to all leg joints of AMOSII through muscle models (Xiong et al., 2014b) (see Supplementary Figure 1).

The local leg control has two components for each leg: (1) a neural forward model transforming the motor signal (efference copy) generated by the CPG into an expected sensory signal for estimating the walking state and (2) elevation and searching control for adapting leg motion (e.g., extension/flexion and elevation/depression).

**Figure 7:**
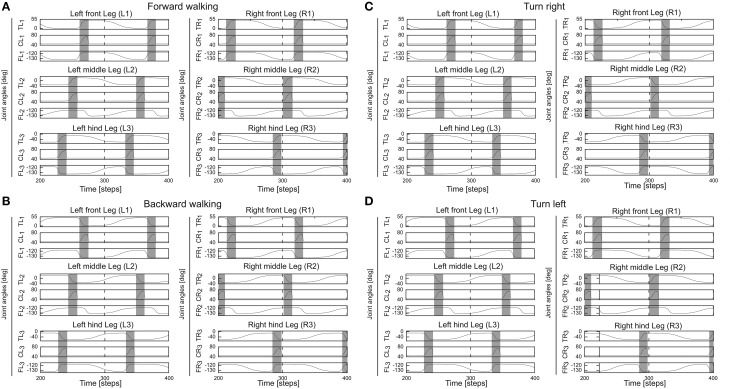
**Angles of the thoraco-coxal (TC-) joint, the coxa-trochanteral (CTr-) joint, and the femur-tibia (FTi-) joint of all legs. (A)** Forward walking. **(B)** Backward walking. **(C)** Turning right. **(D)** Turning left. All joint angles are in degrees. Gray and white areas indicate the swing and stance phases, respectively. Here the CPG generates low frequency periodic signals which lead to a slow wave gait. For this gait, the legs swing one by one from hind to front. This gait is used through out this study. TR1, CR1, FR1 = TC-, CTr-, and FTi-joints of the right front leg (R1); TR2, CR2, FR2 = right middle leg (R2); TR3, CR3, FR3 = right hind leg (R3); TL1, CL1, FL1 = left front leg (L1); TL2, CL2, FL2 = left middle leg (L2); TL3, CL3, FL3 = left hind leg (L3). Note the TC-joint is for forward (+) and backward (−) movements, the CTr-joint is for elevation (+) and depression (−) of the leg, and the FTi-joint is for extension (+) and flexion (−) of the tibia. Further details see main text and Supplementary Material.

The backbone joint control is based on a recurrent neural network consisting of five input neurons, a hidden, postprocessing neuron and an output neuron which drives the backbone joint (see Supplementary Figure 1). All neurons of the locomotion control network are modeled as discrete-time non-spiking neurons. They are updated with a frequency of approximately 27 Hz. The complete neural circuit, which combines the adaptive neural sensory processing network and the modular neural locomotion control network, is shown in Supplementary Figure 1. For obstacle avoidance, the outputs *O*_1(*left*), 2(*right*)_ of the adaptive neural sensory processing network (Figure 2) are transmitted as descending steering signals to the VRNs. This way, AMOSII will walk according to the outputs *O*_1, 2_ (see Table 1). Figure 7 exemplifies all leg joint movements during forward/backward walking and turning right/left. The complete description of the locomotion control network can be seen at our previous work (Manoonpong et al., 2013).

### 2.3. The biomechanical walking robot AMOSII

The biomechanical walking robot AMOSII is a biologically inspired hardware platform (Figure 1B) having two main components: bio-inspired structures and simulated muscles.

Its structures consist of six identical multi-jointed legs and one backbone joint (BJ). Each leg has three joints (three degrees of freedom): the thoraco-coxal (TC-) joint enables forward (+) and backward (−) movements, the coxa-trochanteral (CTr-) joint enables elevation (+) and depression (−) of the leg, and the femur-tibia (FTi-) joint enables extension (+), and flexion (−) of the tibia. The morphology of this multi-jointed leg is modeled on the basis of a cockroach leg but the tarsus segments are ignored. The BJ is inspired by the segmented body joints of a cockroach. It can lean downwards to a minimum position of −45° which is comparable to a cockroach. The joint also allows to lean upwards to a maximum position of +45°. The leaning upward and downward motions are used for climbing over an obstacle. All joints are driven by standard servomotors.

AMOSII has all in all 19 motors and various sensors, e.g., two ultrasonic sensors, a camera, a laser range finder, etc. The ultrasonic sensors act as antennae to detect obstacles. They are used to provide sensory inputs to our adaptive sensory processing network. The camera is used for terrain classification and the laser range finder is used to measure obstacle height in order to distinguish between a wall and a surmountable obstacle (see Kesper et al., 2013; Zenker et al., 2013 for more details). We use a Multi-Servo IO-Board (MBoard) to digitize all sensory input signals (except the camera and laser range finder signals) and generate a pulse-width-modulated signal to control servomotor position. For the robot walking experiments in this study, the MBoard was connected to a personal computer on which the neural locomotion controller was implemented. The update frequency was 27 Hz. Electrical power supply was provided by batteries: one 11.1 V lithium polymer 3200 mAh for all servomotors and two 11.1 V lithium polymers 910 mAh for the electronic board (MBoard) and all sensors (see Manoonpong et al., 2013 for more details). Besides the bio-inspired body and leg structures, AMOSII also has muscle-like mechanisms (called virtual agonist-antagonist mechanisms Xiong et al., 2014b) for variable compliant leg motions. This biomechanical function allows it to achieve stable and energy-efficient locomotion on different surfaces (Xiong et al., 2014a). The complete description of AMOSII can be seen at our previous work (Manoonpong et al., 2013).

## 3. Results

In the previous sections, we describe an adaptive embodied neural closed-loop control system for versatile and adaptive behaviors (Figure 1A). The system consists of neural mechanisms (i.e., the adaptive neural sensory processing network and the modular neural locomotion control network), biomechanics (i.e., the biomechanical walking robot AMOSII with bio-inspired morphology and muscle models), and the environment. Besides, we present the performance and neural dynamics of the adaptive processing network and compare them with the ones of a nonadaptive sensory processing network. Here, we further evaluate the performance of the adaptive embodied system through three main experiments.

The first experiment investigates the synaptic changes and neural outputs of the adaptive neural sensory processing network as well as robot behavior in a very complex environment with many obstacles, different sharp corners, and narrow passages in simulation. In this experiment, we initialized all synaptic weights to zero and let the robot start from a certain location in the environment. The experimental result is shown in Figures 5F,G. It can be seen that the learning mechanism can stably adapt the weights such that the robot can walk around and adapt its turning angle for avoiding obstacles in different situations. The adaptation also enables the robot to effectively escape from sharp corners or deadlock (different to a static network; Figures 3F,G). Consequently, the robot can successfully explore and navigate in the complex environment.

**Figure 8:**
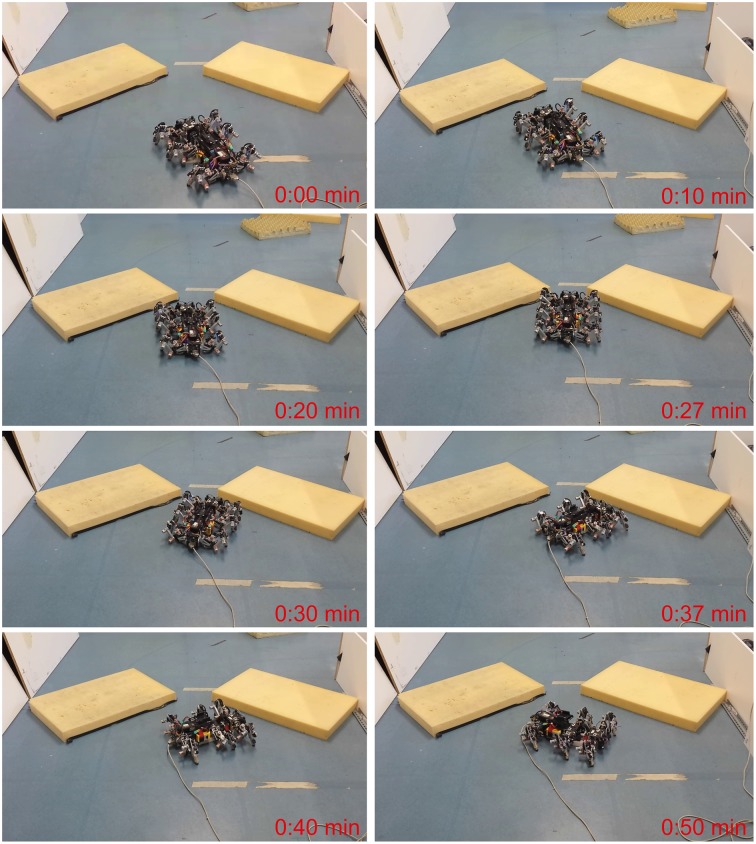
**Escape behavior of AMOSII from a narrow passage**. Snapshots of AMOSII during interaction with the environment. We encourage readers to also see the video of this experiment at Supplementary Video 1.

The second experiment presents real robot behaviors in two different environments. The first environment follows the one shown in Figure 5B. The experimental result is shown as a series of photos of AMOSII (Figure 8). AMOSII first walked toward a narrow passage (0:00 min). Then, it detected the obstacle on its left (0:10 min), thereby, turning to the right (0:20 min). It got stuck briefly in front of the gap (0:27 min) because both sensors detected both obstacles. In this situation, the synaptic weights of the left neuron of the adaptive sensory processing network were strongly modified compared to the synaptic weights of the right neuron. This adaptation reinforced the activation of the left neuron while inhibiting the right one. As a consequence, AMOSII could continually turn to the right (0:30–0:40 min) and then successfully escaped from the passage (0:50 min). In contrast, AMOSII got stuck and failed to escape from it when the non-adaptive network was used (described in Section 2.1.1; see Supplementary Video 1).

**Figure 9:**
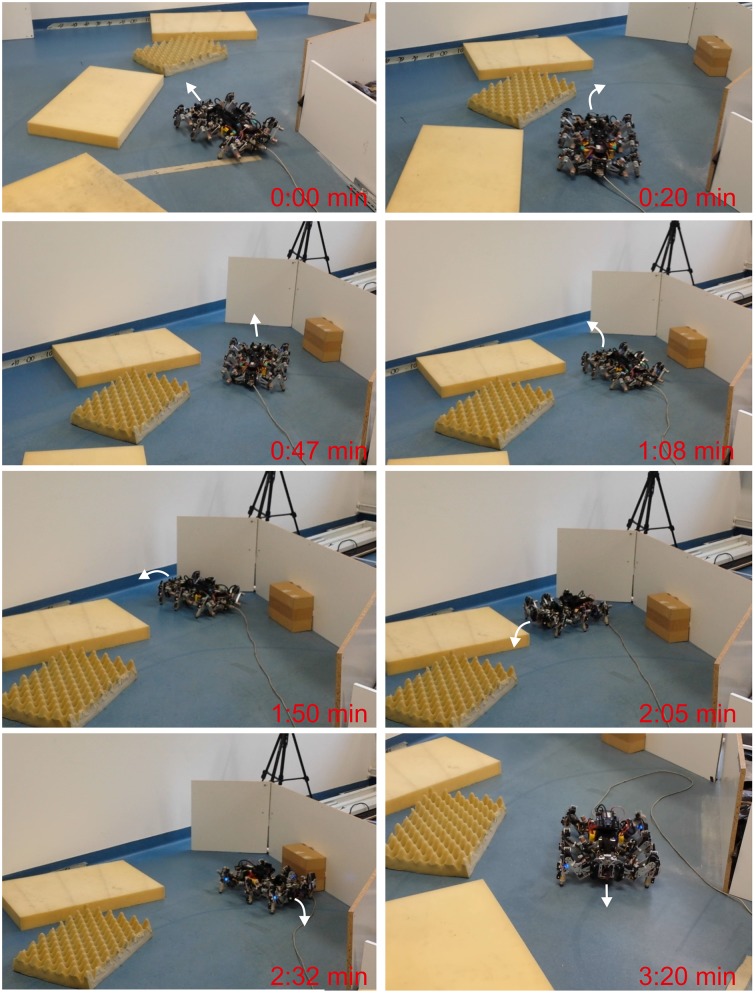
**Navigation of AMOSII in a complex path**. Snapshots of AMOSII during interaction with the environment. We encourage readers to also see the video of this experiment at Supplementary Video 2.

The second environment was constructed as a complex path consisting of side walls on the right, small obstacles on the left, and deadlock at the end of the path. This experiment aims to access only obstacle avoidance behavior while walking in the path. Thus, only the two ultrasonic sensors were used to allow AMOSII to detect obstacles while the laser range finder for detecting obstacle height was ignored. The experimental result is shown as a series of photos of AMOSII (Figure 9). AMOSII first entered the path (0:00 min) and then turned to the right since it detected an obstacle on the left (0:20 min). It then continued to walk toward the end of the path (0:47 min) and approached it (1:08 min). Afterwards, AMOSII turned left to avoid the deadlock (1:50–2:05 min), turned right to avoid the left wall (2:32 min), and finally went out of the path (3:20 min). This successful navigation was autonomously controlled by the descending steering signals from the adaptive neural sensory processing network. In contrast, AMOSII got stuck in the path when the non-adaptive network (described in Section 2.1.1) was employed (see Supplementary Video 2).

**Figure 10:**
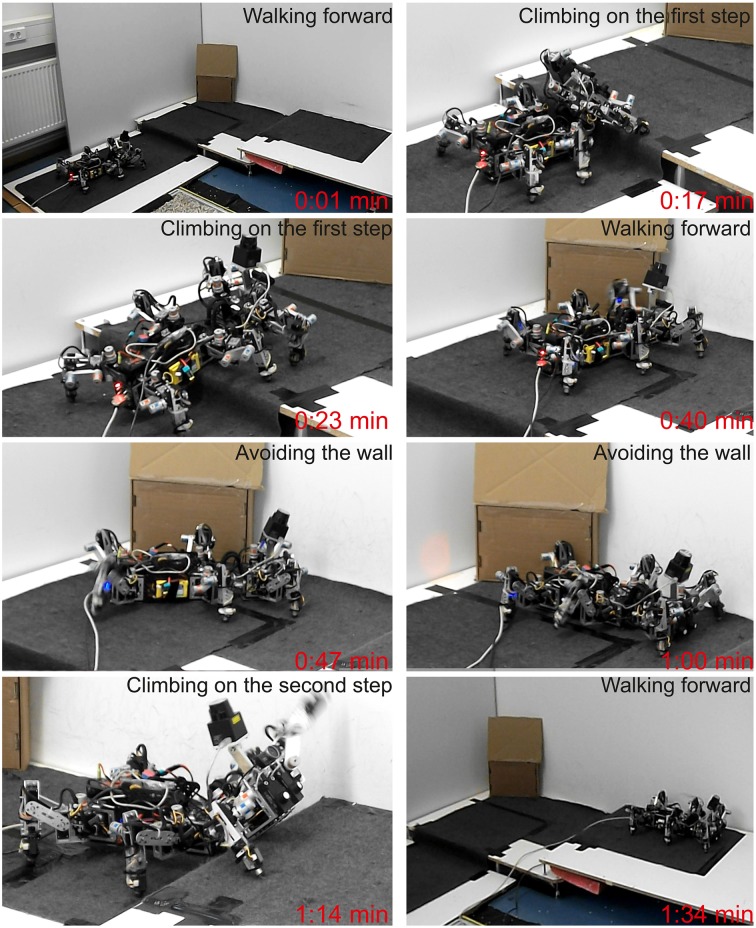
**Versatile behavior of AMOSII in a complex environment**. Snapshots of AMOSII during interaction with the environment. We encourage readers to also see the video of this experiment at Supplementary Video 3.

The final experiment demonstrates versatile behavior (walking, climbing up steps, and avoiding a wall) in a complex environment. To do so, we constructed a track consisting of a ground floor and two elevated platforms and let AMOSII start to walk from the first platform (ground floor). In this experiment, we used both the ultrasonic sensors and the laser range finder to allow AMOSII to detect obstacles and distinguish between climbable (i.e., step) and non-climbable (e.g., wall) obstacles. If AMOSII detects a climbable obstacle, it will start to climb over it using extra BJ control embedded in the neural locomotion control circuit (Goldschmidt et al., 2014, see also Supplementary Figure 1). In contrast, if it detects a non-climbable obstacle, it will avoid it. The avoiding behavior is driven by the adaptive sensory processing network. The experimental result is shown as a series of photos of AMOSII (Figure 10). It can be seen that AMOSII started walking toward the first platform (0:01 min). It then detected the platform as a climbable obstacle and climbed onto it (0:17–0:23 min). Afterwards, AMOSII continued to walk on the first platform (0:40 min). While walking on it, AMOSII detected the wall in front of it, thereby, turning to avoid the wall (0:47–1:00 min). After avoiding the wall, AMOSII climbed onto the second platform (1:14 min) and continued to walk on it (1:34 min). The versatile behavior was achieved by the combination of the complete neural circuit (Supplementary Figure 1) and the bio-inspired structure of AMOSII (Figure 1B).

## 4. Discussion

In this study, we introduced neural mechanisms for versatile and adaptive behaviors and used our biomechanical walking robot AMOII as an experimental platform to evaluate the performance of the mechanisms and demonstrate the behaviors. The mechanisms consist of adaptive neural sensory processing and modular neural locomotion control. The sensory processing was formed by a two-neuron recurrent network with fully connections. Online correlation-based learning with synaptic scaling was employed to stably modify network connections. In principle, this online learning mechanism can increase or decrease the weights of the connections depending on the interaction of the robot with its environment; thereby changing neural dynamics in the network (e.g., from hysteresis effects to a stable attractor and vice versa). This changing neural dynamics can be exploited for generating different turning angles including very large ones to avoid different obstacles and corners. Specifically, the sensory preprocessing takes exteroceptive sensory inputs provided by, for instance, two ultrasonic sensors installed at the front of AMOSII and translates the signals into descending steering commands to the locomotion control to generate adaptive turning angles with short-term memory when facing to obstacles, sharp corners, or narrow passages. This results in adaptively avoiding obstacles and escaping from corners/deadlock and narrow passages. Furthermore, this also enables AMOSII to successfully explore and navigate in cluttered unknown environments.

Several obstacle avoidance techniques have been developed in the past (Pasemann et al., 2003; Harter and Kozma, 2005; Vargas et al., 2009; Risi and Stanley, 2012; Pitonakova, 2013). A classical way is to use Braitenberg control (Braitenberg, 1986) which reactively controls an agent with respect to the activations of sensory inputs. For this approach, the agent will turn as long as it detects an obstacle. At a corner or deadlock, it might switch between turning left and right several times to avoid or escape from the situation or it sometimes gets stuck. To overcome the problem, a sensor array can be used (Fend et al., 2003; Dongyue et al., 2013; Mohammad et al., 2013) together with short-term memory (Hülse and Pasemann, 2002). Hülse and Pasemann (2002) developed a minimal recurrent controller (MRC) using an evolutionary algorithm (Hülse et al., 2004). The MRC consists of two neurons with mutual inhibitory synapses between the neurons and a self-excitatory synapse of each neuron. According to the recurrent connections, the controller exhibits hysteresis effects which act as short-term memory to prolong turning action. The MRC was tested on a simple Khepera robot, a two wheeled platform (2 DOFs) with a sensor array (i.e., six front proximity sensors). While the robot can effective avoid obstacles, it has sometimes difficulties to explore and avoid obstacles in complex environments with many obstacles, sharp corners, and narrow passages. This is because MRCs exhibit three hysteresis effects (Hülse and Pasemann, 2002) which can be switched among them by inputs. This leads to a certain degree of turning angle which sometimes is not enough for avoiding, for instance, a sharp corner. Note that turning angle or turning duration is basically derived from the width of the hysteresis. According to this, Toutounji and Pasemann (2014) introduced short-term plasticity induced by self-regulating neurons (Zahedi and Pasemann, 2007) in MRC. This allows the wheel-driven robot ALICE with five distance sensors to capable of avoiding sharp corners. However, in this approach, different to the present one, the activity states have to be predefined.

In contrast to the previous approaches, our work here show that the small adaptive preprocessing unit with only two sensors allow a many degrees of freedom machine (like AMOSII) to learn and adapt its behavior to successfully avoid obstacles and navigate in a very complex environment. However, it is important to note that the agent does not learn ones the complete environment. It basically adapts to the current situation it is situated in, moves on, and adapts to the next situation. The adaptation time needed for each situation cannot be precisely estimated as this depends on several parameters (entrance angle, gap between objects, number of objects, etc.) which can already seen in Figure 5. Further investigations are needed which are beyond the scope of this study.

In addition to the preprocessing unit, the neural locomotion control can also allow AMOSII to walk around and climb over a climbable obstacle. The plasticity mechanism used here is different from others as it relies on a dynamic adaptation whereby synaptic strengths are memorized with respect to the neuronal excitation (Tetzlaff et al., 2011). Thereby, the presented mechanism allows the neural dynamics to change from hysteresis effects to single fixed point states (lower and upper) in order to keep the neural output stay for longer duration at certain values. This then generates, for instance, a very large turning angle for the robot which will be sufficient to avoid or escape from sharp corners and narrow passages. Once the robot has escaped from the situation; i.e., its sensory inputs become inactive, the synaptic strengths will decay. Thus, the neural dynamics will change from having only the upper fixed point to having also the lower fixed point (hysteresis). For survival behavior, it is in the interest of the agent to keep the excitation of the front ultrasonic distance sensors close to minimum. The agent has a stable moving forward behavior, this is only interrupted by approaching obstacles. Here the stable forward behavior is interrupted and leads into a stable turning behavior of the agent. As soon as the excitation ends, the adaptive preprocessing unit generates negative steering motor outputs. Thus, the agent continues with the stable forward behavior. The effective control and timing of the adaptive processing network depends also on the chosen learning and forgetting parameters, which were currently picked by hand. Different values of these parameters can reflect different learning speeds or different timescales of adaptivity. The current chosen values deliver fast adaptation in difficult situations, but also enough stability to prevent the hysteresis to fall into an infinity-feedback-loop. These parameters seem to be quite critical for satisfactory behavior. However, they can be found in principle by using evolutionary algorithms.

Taken together this work provides a further step suggesting how neural dynamics, plasticity, sensory feedback, and biomechanics can be combined to generate versatile and adaptive behaviors of complex robots. While biomechanical components (e.g., structures and muscles) allow for complex movements (walking, climbing), neural dynamics and plasticity embedded in sensory processing and control networks as well as sensory feedback form coordination, generate locomotion, and provide adaptation. The results presented here show that the employed embodied adaptive neural closed-loop system (Supplementary Figure 1) is a powerful approach for achieving versatility and adaptivity in machines. As the neural mechanisms are modular, it is flexible and offers the future possibility of integrating other modules, like a goal-directed navigation learning module (Zeidan et al., 2014) and a neural path integration module (Goldschmidt et al., 2015). This will enable the robotic system to be capable of navigating in complex environments toward given goals and autonomously return to its home position. It is important to emphasize that although here we show the use of the adaptive preprocessing network for a walking robot system, the network can be applied to other mobile robot systems for generating adaptive obstacle avoidance.

### Conflict of interest statement

The authors declare that the research was conducted in the absence of any commercial or financial relationships that could be construed as a potential conflict of interest.
