# Supplementary material for: Synaptic plasticity in a recurrent neural network for versatile and adaptive behaviors of a walking robot
Source: Front Neurorobot. 2015 Oct 13;9:11. doi: 10.3389/fnbot.2015.00011 (PMC4602151; doi:10.3389/fnbot.2015.00011)
Supplement: Supplementary file 1 [file DataSheet1.PDF]

Supplementary information accompanying the manuscript
Synaptic plasticity in a recurrent neural network for versatile and adaptive behaviors of a walking robot

This supplementary information includes:

1) Complete Neural Circuit

2) Input-output Analysis of the Inhibitory Synapses of a Recurrent Neural Network

3) Supplementary Figures 1-2

4) Supplemental Video Legends 1-3

1 Complete Neural Circuit

Supplementary Figure 1 shows the complete circuit for complex behavior generation. It consists of sensory processing, modular neural locomotion control, and muscle models. It receives sensory signals and processes them through sensory processing modules. The processed sensory signals are transmitted to a modular neural locomotion control network consisting of three main mechanisms: Central pattern generator(CPG)-based control (modules I-IV), local leg control (modules V-VI), and backbone joint control (module VII). The locomotion control network basically coordinates all leg and body joints and generates their movements through muscle models which allow for compliance **Xiong et al.** (2014). It also at the same time uses the processed sensory signals to adapt the movements to deal with cluttered unknown environments and uneven terrains.

Module I (CPG with neuromodulation): MI = modulatory input; $C_{1,2}$ = output neurons of the CPG. We use a hyperbolic tangent (tanh) transfer function for the CPG neurons.

Module II (neural CPG postprocessing): $CP_{1,2}$ = postprocessing neurons with a step function; $Int_{1,2}$ = integrator units.

Module III (neural motor control): $I_{1,\ldots,4}$ = neural control parameters for generating different walking directions and stopping motion; $H_{1,\ldots,14}$ = interneurons of the phase switching network (PSN); $H_{15,\ldots,28}$ = interneurons of the velocity regulating networks (VRNs). We use a tanh transfer function for the interneurons. Parameters are $A = 1.7246, B = -2.48285, C = -1.7246$.

Module IV (motor neurons): $M_{1,\ldots,5}$ = premotor neurons; TR_1, CR_1, FR_1 = TC-, CTr- and FTi-motor neurons of the right front leg (R1); TR_2, CR_2, FR_2 = right middle leg (R2); TR_3, CR_3, FR_3 = right hind leg (R3); TL_1, CL_1, FL_1 = left front leg (L1); TL_2, CL_2, FL_2 = left middle leg (L2); TL_3, CL_3, FL_3 = left hind leg (L3); BJ = a backbone motor neuron which is controlled by the backbone joint control (module VII); τ = ipsilateral lag (i.e., 16 time steps or $\approx$ 0.6 s); τ_L = the phase shift between both left and right sides (i.e., 48 time steps or $\approx$ 2 s). We use piecewise linear transfer functions for the premotor and motor neurons.

Module V (adaptive neural forward models): $F_{1,\ldots,6}$ = adaptive hysteresis neurons for motor signal transformation; W_I, W_R, B = learning parameters; $P_{1,\ldots,6}$ = postprocessing neurons; Δ = an error between the expected foot contact signal and the actual one. We use a tanh transfer function for the hysteresis and postprocessing neurons.

Module VI (searching and elevation control): $PD_{1,\ldots,6}$ = preprocessing neurons which provide only a positive error ($+\Delta$); $ND_{1,\ldots,6}$ = preprocessing neurons which provide only a negative error ($-\Delta$); $E_{1,\ldots,6} = S_{1,\ldots,6}$ = recurrent neurons (i.e., accumulators). We use piecewise linear transfer functions for the preprocessing neurons and use a linear transfer function for the recurrent neurons.

Module VII (backbone joint control): $BJ_{1,\ldots,6}$ = five input neurons and one hidden neuron with a linear threshold transfer function. BJ_7 = output neuron of the backbone joint control.

Note that in all modules, all numbers are synaptic weights and the ones marked with subscript "B" refer to fixed bias terms (see **Manoonpong et al.** (2013); **Goldschmidt et al.** (2014) for details of all these modules).

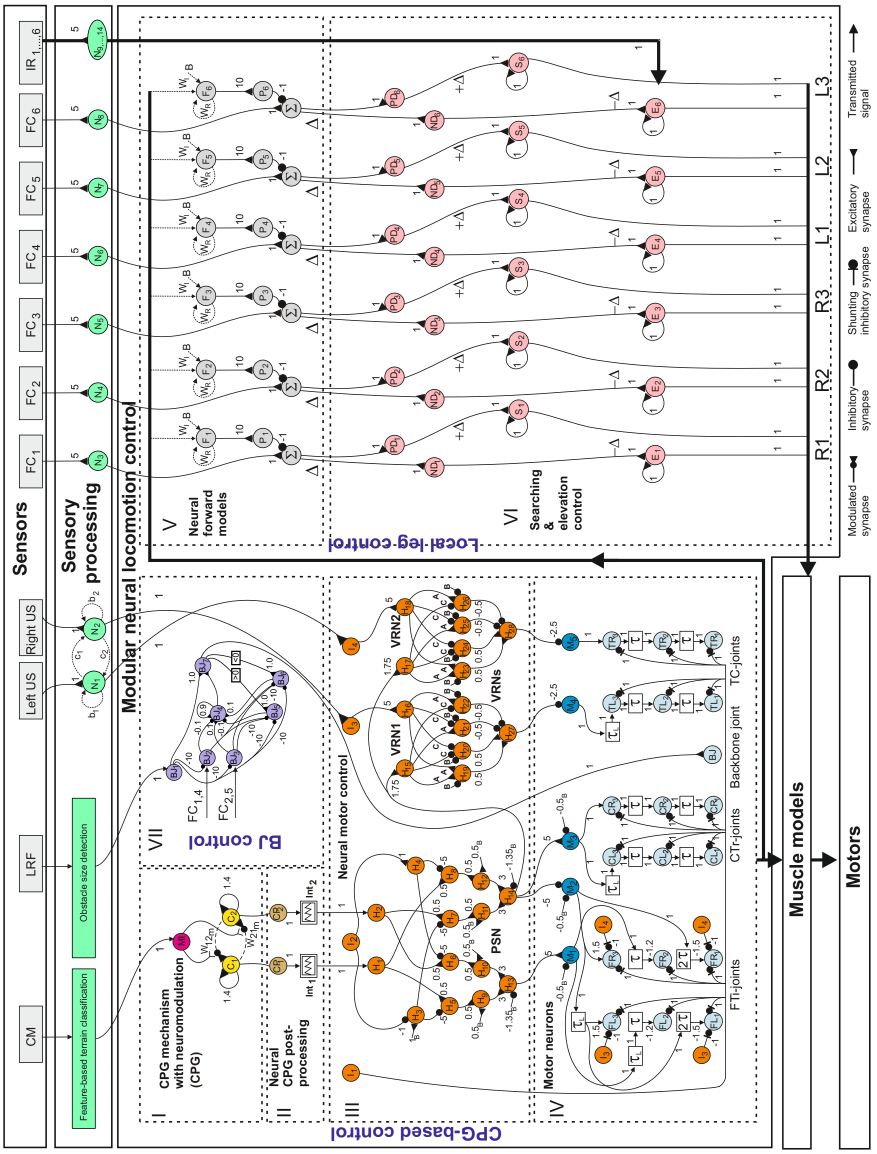

Supplementary Figure 1: Complete circuit for the versatile and adaptive behaviors of the biomechanical walking robot AMOSII (see text for details).

Different exteroceptive and proprioceptive sensors can be used as inputs to the controller to generate stimulus induced behavior and adaptive locomotion. The sensors are: one USB camera (CM), one laser range finder (LRF), left and right ultrasonic sensors (US), six foot contact sensors ($FC_{1,...,6}$), six infrared reflex sensors ($IR_{1,...,6}$). All raw sensory signals are preprocessed using neural preprocessing except the visual signal and the laser range finder signal which are done by using an online feature-based terrain classification algorithm **Zenker**

et al. (2013) and an obstacle height detection algorithm **Kesper et al.** (2013), respectively.

We use two neurons ($N_{1,2}$) with a tanh transfer function to form the adaptive neural processing network of the left and right US signals. The network outputs provide orienting control signals which are transmitted to $I_{3,4}$ of the neural motor control module. As a result, AMOSII can effectively perform an appropriate turning angle to avoid obstacles, narrow passages, or corners.

We use neurons ($N_{3,...,14}$) with a tanh transfer function for preprocessing the $\mathrm{FC}_{1,...,6}$ and $\mathrm{IR}_{1,...,6}$ signals. This is because the sensor signals contain small noise which can be eliminated by the nonlinearity of the neuron. The preprocessed sensor signals are used for local leg control **Manoonpong et al.** (2013).

The muscle models consists of a pair of agonist and antagonist mechanisms (also called virtual agonistantagonist mechanism (VAAM)). It produces active and passive forces using its contractile and parallel elements (CEs and PEs). Here each joint of AMOSII is driven by a pair of the VAAM. Virtual means that the joint, physically driven by a standard servo motor, imitates muscle-like behaviors as if it were driven by a pair of physical agonist and antagonist muscles. The joint actuation relies on the CEs while the joint compliance is achieved by the PEs. The CEs are approximated by the neural outputs of the modular neural locomotion control and the PEs are modeled as spring-damper systems with stiffness (K) and damper (D) parameters. Changing the parameters (K, D) enables AMOSII to achieve variable compliant leg motions, thereby leading to adaptive and energy-efficient walking on different terrains (not shown here but see **Xiong et al.** (2014)).

2 Input-output Analysis of the Inhibitory Synapses of a Recurrent Neural Network

Here, we present the input-output relation for different strengths of inhibition of a recurrent neural network (see **Fig. 2** of the main text). We analyze the inhibitory influence by setting the excitatory weights to a constant value ($b_1 = b_2 = 2.4$) and varied the input I_2 and the inhibitory weight ($c_1 = c_2 \equiv c$). For each of these sets of parameters we changed the first input I_1 and measured the corresponding outputs O_1 and O_2 (see **Supplementary Figure 2**).

By varying the input I_1, we see that the output O_1 passes through a hysteresis while O_2 is basically uneffected. Changing the second input I_2 (columns) brings the output O_2 to a different baseline and the onset of the hysteresis of O_1 changes slightly. Now, for increasing the inhibitory weight c (rows) we see that the onset as well as the width of the hysteresis changes. However, these dynamics could become even more complex (see, for instance, $c = -3.5; I_2 = 0$). Thus, we expect that adapting inhibition could imply even more complex behaviors which have to be analyzed in further detail. However, this is beyond the scope of this manuscript. Here, it is behaviorally important that inhibition uncorrelates the output (O_2 is uneffect by changes of I_1) and that, as for excitatory plasticity, the parameters of the hysteresis are changed.

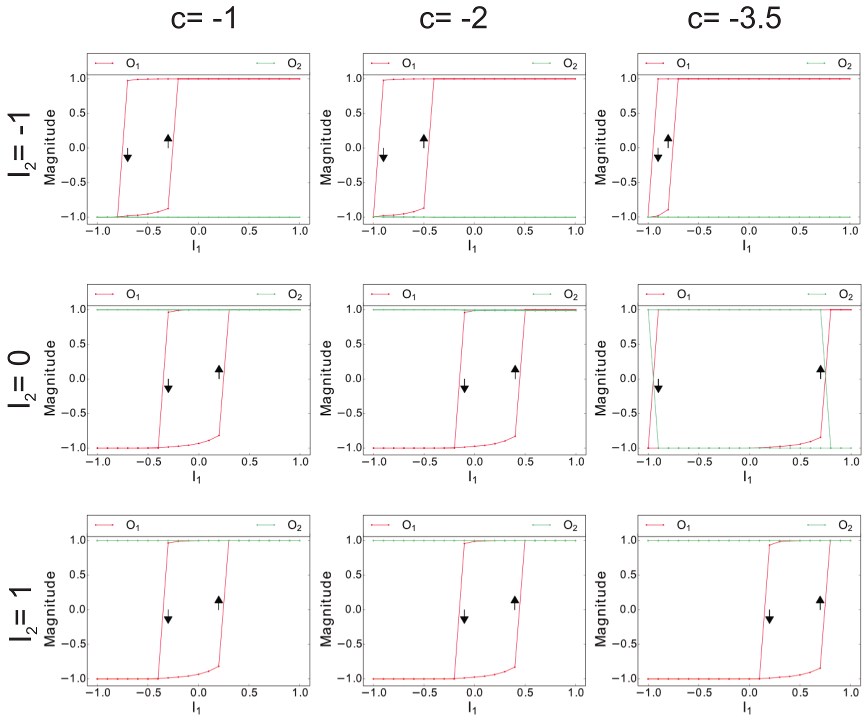

Supplementary Figure 2: **Analysis of the input-output relation for different strengths of inhibition. Parameters:** $b_1 = b_2 = 2.4$, $c_1 = c_2 = c$ **(see text for details).**

Supplemental Video Legends

Supplementary Video 1: **Comparison of escape behavior of AMOSII from a narrow passage using the non-adaptive and adaptive networks**. AMOSII got stuck and failed to escape from the narrow passage when the non-adaptive network was used while it can successfully escape from the situation when the adaptive network was used.
(http://manoonpong.com/Frontiers/2015/SupplementaryVideo1.wmv)

Supplementary Video 2: **Comparison of navigation of AMOSII in a complex path using the non-adaptive and adaptive networks**. AMOSII got stuck in the path when the non-adaptive network was used while it performed successful navigation when the adaptive network was used.
(http://manoonpong.com/Frontiers/2015/SupplementaryVideo2.wmv)

Supplementary Video 3: **Versatile behavior of AMOSII in a complex environment**. AMOSII can autonomously walk, climb up steps, and avoid a wall in the complex environment.
(http://manoonpong.com/Frontiers/2015/SupplementaryVideo3.wmv)

References

Goldschmidt, D., Wörgötter, F., and Manoonpong, P. (2014), Biologically-inspired adaptive obstacle negotiation behavior of hexapod robots, *Front. Neurorobot.*, 8, 3, doi: 10.3389/fnbot.2014.00003

Kesper, P., Grinke, E., Hesse, F., Wörgötter, F., and Manoonpong, P. (2013), Obstacle/gap detection and terrain classification of walking robots based on a 2d laser range finder, in 16th International Conference on Climbing and Walking Robots and the Support Technologies for Mobile Machines (CLAWAR), 419–426

Manoonpong, P., Parlitz, U., and Wörgötter, F. (2013), Neural control and adaptive neural forward models for insect-like, energy-efficient, and adaptable locomotion of walking machines, *Frontiers in neural circuits*, 7, 12, doi: 10.3389/fncir.2013.00012

Xiong, X., Wörgötter, F., and Manoonpong, P. (2014), Virtual agonist-antagonist mechanisms produce biological muscle-like functions: An application for robot joint control, *Industrial Robot: An International Journal*, 41, 4, 340–346

Zenker, S., Aksoy, E. E., Goldschmidt, D., Worgotter, F., and Manoonpong, P. (2013), Visual terrain classification for selecting energy efficient gaits of a hexapod robot, in Advanced Intelligent Mechatronics (AIM), 2013 IEEE/ASME International Conference on (IEEE), 577–584
